# Prospective function of FtsZ proteins in the secondary plastid of chlorarachniophyte algae

**DOI:** 10.1186/s12870-015-0662-7

**Published:** 2015-11-10

**Authors:** Yoshihisa Hirakawa, Ken-ichiro Ishida

**Affiliations:** Faculty of Life and Environmental Sciences, University of Tsukuba, 1-1-1 Tennodai, Tsukuba, Ibaraki 305-8572 Japan

**Keywords:** Chlorarachniophytes, Endosymbiosis, FtsZ, Nucleomorph, Plastid division

## Abstract

**Background:**

Division of double-membraned plastids (primary plastids) is performed by constriction of a ring-like division complex consisting of multiple plastid division proteins. Consistent with the endosymbiotic origin of primary plastids, some of the plastid division proteins are descended from cyanobacterial cell division machinery, and the others are of host origin. In several algal lineages, complex plastids, the “secondary plastids”, have been acquired by the endosymbiotic uptake of primary plastid-bearing algae, and are surrounded by three or four membranes. Although homologous genes for primary plastid division proteins have been found in genome sequences of secondary plastid-bearing organisms, little is known about the function of these proteins or the mechanism of secondary plastid division.

**Results:**

To gain insight into the mechanism of secondary plastid division, we characterized two plastid division proteins, FtsZD-1 and FtsZD-2, in chlorarachniophyte algae. FtsZ homologs were encoded by the nuclear genomes and carried an N-terminal plastid targeting signal. Immunoelectron microscopy revealed that both FtsZD-1 and FtsZD-2 formed a ring-like structure at the midpoint of bilobate plastids with a projecting pyrenoid in *Bigelowiella natans*. The ring was always associated with a shallow plate-like invagination of the two innermost plastid membranes. Furthermore, gene expression analysis confirmed that transcripts of *ftsZD* genes were periodically increased soon after cell division during the *B. natans* cell cycle, which is not consistent with the timing of plastid division.

**Conclusions:**

Our findings suggest that chlorarachniophyte FtsZD proteins are involved in partial constriction of the inner pair of plastid membranes, but not in the whole process of plastid division. It is uncertain how the outer pair of plastid membranes is constricted, and as-yet-unknown mechanism is required for the secondary plastid division in chlorarachniophytes.

**Electronic supplementary material:**

The online version of this article (doi:10.1186/s12870-015-0662-7) contains supplementary material, which is available to authorized users.

## Background

Plastids are endosymbiotically derived organelles, and their evolutionary histories are complicated due to multiple endosymbiotic events. Plants and three algal groups (green algae, red algae, and glaucophytes) acquired plastids through a single primary endosymbiosis between a heterotrophic protist and a photosynthetic cyanobacterium, and we refer to the plastids originating from this process as primary plastids [1, 2]. In contrast, many other algae (e.g., dinoflagellates, heterokonts, haptophytes, cryptophytes, euglenophytes, and chlorarachniophytes) and apicomplexan parasites possess complex plastids. These are called secondary plastids, which evolved through endosymbiotic uptakes of red and green algae by distinct protists [3, 4]. Multiple secondary endosymbioses led to the phylogenetic diversity of the current plastid-bearing organisms [5]. In terms of structures, primary and secondary plastids are distinct in the number of envelope membranes. Primary plastids are surrounded by two membranes, whereas secondary plastids have one or two additional membranes; euglenophyte and dinoflagellate plastids are bounded by three membranes, and the others have four membranes [6]. In four membrane-bound secondary plastids, the two innermost membranes are regarded as descendants of the primary plastid membranes of the engulfed alga, and the two outermost membranes are suggested to be the former phagosomal membrane of the host and the relict plasma membrane of the endosymbiont, respectively [6, 7]. The outermost membrane is connected to the endoplasmic reticulum (ER) in some secondary plastids of red algal origin, and the recent ER-enclosure model has proposed that the outer pair of membranes are derived from the host ER [8]. These additional membranes should be a barrier for transport of nucleus-encoded plastid proteins as well as for metabolite exchange. Thus, secondary plastid-bearing organisms had to evolve transport mechanisms for breaking through the membranes [9, 10]. Furthermore, in terms of plastid division, new mechanisms would be necessary for constriction of the additional membranes in secondary plastids.

Primary plastid division is performed by the simultaneous constriction of the two envelope membranes at the division site, which is mediated by a ring-like division complex encompassing both the inside and the outside of the two membranes [11, 12]. Consistent with the origin of primary plastids, protein components of the division complex on the stromal side are typically descended from cyanobacterial cell division proteins, and several host-derived proteins comprise the cytoplasmic portion [13]. In plants and algae, prior to the onset of plastid division, the tubulin-like GTPase FtsZ assembles into a ring-like structure on the stromal surface of the inner plastid membrane at the division site [14–18]. In the model plant *Arabidopsis thaliana*, the FtsZ ring (Z ring) is tethered to the inner plastid membrane via an interaction with the integral membrane protein ARC6 [19], and the positioning of the Z ring is regulated by several stromal proteins, MinD [20], MinE [21], ARC3 [22], and MCD1 [23]. Subsequently, the stromal plastid division proteins directly or indirectly recruit cytoplasmic components of the division complex, including the outer membrane proteins PDV1 and PDV2 [24], and the dynamin-related GTPase DRP5B (also called ARC5) that assembles into a ring-like structure on the cytoplasmic portion [25]. Two self-assembling GTPases, FtsZ and DRP5B, are thought to generate contractile force for membrane constriction [26, 27]. In earlier electron microscopic studies, electron-dense ring structures, called the plastid-dividing (PD) rings, were observed at the plastid division site on both the stromal and the cytoplasmic surface of the plastid membranes in plants and algae [28]. A recent study showed that the outer PD ring of the primitive red alga *Cyanidioschyzon merolae* is composed of polyglucan filaments [29], and filament sliding mediated by DRP5B (CmDnm2) may generate a contractile force for membrane constriction [30]. The principal feature of the plant-like plastid division mechanism seems to be widely conserved in primary plastid-bearing algae that have homologs of plastid division proteins [31]. However, some plastid division proteins are depleted in certain algal lineages [13, 32]. For example, the glaucophyte *Cyanophora paradoxa* genome does not encode any known plastid division proteins of host origin, and constriction of the outer membrane appears to rely on the peptidoglycan hydrolyzing activity with DipM, as in cyanobacterial cell division [33]. The genome of the red alga *C. merolae* encodes only two plant-like plastid division proteins, FtsZ and DRP5B, and it remains unknown how positioning of the plastid division complex at the division site is regulated.

Several homologous genes of primary plastid division proteins have also been identified in whole genomes of secondary plastid-bearing organisms [13]. As shown in Table 1 (updated from [13, 18]), homologous genes for FtsZ, MinD, MinE, and/or DRP5B, were found in the heterokonts *Thalassiosira pseudonana*, *Phaeodactylum tricornutum*, and *Aureococcus anophagefferens*, the haptophyte *Emiliania huxleyi*, and the cryptophyte *Guillardia theta* based on BLAST surveys. These homologous genes are assumed to be derived from the engulfed red algae via secondary endosymbioses. Plastid division proteins are mostly encoded by the nuclear genomes as the result of endosymbiotic gene transfer, whereas the MinD and MinE of *G. theta* and the MinD of *E. huxleyi* are encoded by the plastid genomes [34, 35], and *G. theta ftsZ* gene is found in the nucleomorph genome that is a relict nuclear genome of the red algal endosymbiont [36]. Localization studies on secondary plastid division proteins are limited to a few organisms. Green fluorescent protein (GFP)-tagged experiments confirmed that FtsZ proteins of the diatom *Chaetoceros negogracile* carried an N-terminal plastid targeting signal [37], and immunofluorescence localization revealed that the *T. pseudonana* DRP5B formed a ring-like structure at the putative plastid division site [11]. Thus, constriction of the inner pair of secondary plastid membranes appears to be achieved by a portion of the primary plastid division complex that is derived from the engulfed endosymbiont. Although it is uncertain how the outer pair of secondary plastid membranes are divided, a study of apicomplexan parasites has shed light on this problem. Apicomplexans have non-photosynthetic secondary plastids (called apicoplasts) that are surrounded by four membranes. van Dooren et al. (2009) reported that a dynamin-related protein of *Toxoplasma gondii* (TgDrpA) localized to punctate regions on the apicoplast surface and it was essential for apicoplast division [38]. The *drpA* is an apicomplexan-specific gene that is phylogenetically distinct from the *drp5B* genes of primary plastid-bearing organisms. Interestingly, apicomplexans lack any homologous genes of primary plastid division proteins including FtsZ and DRP5B, suggesting that apicomplexans have lost the primary plastid division machinery and developed a new mechanism mediated by DrpA [38]. To date, few proteins have been characterized as a portion of the mechanism of secondary plastid division, furthermore, the whole picture of secondary plastid division is still unrevealed.

**Table 1:** Distribution of homologous genes for plastid division proteins

organisms	FtsZ	ARC6	MinC	MinD	MinE	DRP5B/ARC5 (DrpA)^a^	PDV, ARC3, MCD1	PDR1
*Synechocystis PCC6803*	BAA17496	BAA10060	BAA10664	BAA10662	BAA10661	N/A	N/A	N/A
*Cyanophora paradoxa*	BAD99307	Contig37232	N/A	BAM09147	BAM09148	N/A	N/A	N/A
*Cyanidioschyzon merolae*	BAC87807,	N/A	N/A	N/A	N/A	BAC55068	N/A	CMR358C
	BAC87808							
*Chlamydomonas reinhardtii*	XP_001702420,	XP_001690917	XP_001692722	XP_001697031	XP_001697195	XP_001702662	N/A	N/A
	XP_001700508							
*Arabidopsis thaliana*	At2g36250,	At5g42480	N/A	At5g24020	At1g69390	At3g19720	At5g53280 ^(PDV)^	N/A
	At3g52750,						At2g16070 ^(PDV)^	
	At5g55280						At1g75010 ^(ARC3)^	
							At1g20830 ^(MCD1)^	
*Thalassiosira pseudonana*	XP_002296337,	N/A	N/A	XP_002291334	N/A	XP_002290986	N/A	N/A
	XP_002294718,							
	XP_002293505							
*Phaeodactylum tricornutum*	XP_002182929,	N/A	N/A	N/A	N/A	XP_002181459	N/A	N/A
	XP_002182240,							
	XP_002185124							
*Aureococcus anophagefferens*	XP_009032511,	N/A	N/A	N/A	N/A	XP_009032466	N/A	N/A
	XP_009038601							
*Guillardia theta*	XP_001713182^Nm^	N/A	N/A	NP_050687^P^	NP_050686^P^	N/A	N/A	N/A
*Emiliania huxleyi*	EOD19496,	N/A	N/A	YP_277381^P^	EOD04187	EOD14983,	N/A	N/A
	EOD06732					EOD23222		
*Toxoplasma gondii*	N/A	N/A	N/A	N/A	N/A	(FJ264918)^a^	N/A	N/A
*Bigelowiella natnas*	JGI39262,	N/A	N/A	N/A	N/A	N/A	N/A	N/A
	JGI92991							

The presence/absence of genes are represented by accession numbers of the NCBI GenBank, JGI, or the *Cyanophora paradoxa* Genome Project. N/A represents ‘not applicable’. The data is updated from [13, 18]

^Nm^nucleomorph-genome encoded

^P^plastid-genome encoded

^a^apicomplexan-specific dynamin-related protein

To gain insight into secondary plastid division mechanisms, we investigated homologs of primary plastid division proteins in chlorarachniophyte algae that obtained secondary plastids by the uptake of a green alga. Chlorarachniophyte plastids are surrounded by four membranes. Each plastid possesses a relict nucleus (called nucleomorph) of the endosymbiont in the periplastidal compartment between the inner and the outer pair of membranes, and the existence of the nucleomorph would make plastid division more complicated [39]. Genome sequencing has been completed in the chlorarachniophyte *Bigelowiella natans*, including the nuclear genome [40], nucleomorph [41], and plastid [42]. A previous proteomic study identified two nucleus-encoded FtsZ homologs in isolated *B. natans* plastids [43]. In this study, we report detailed subcellular localization and periodic gene expression pattern for two FtsZ proteins, named FtsZD-1 and FtsZD-2, in the chlorarachniophyte *B. natans*. Our findings suggest that the FtsZD proteins are involved in partial constriction of the two innermost plastid membranes, but not in the whole process of plastid division. It is uncertain how the outer pair of plastid membranes is constricted, and as-yet-unknown components are required for secondary plastid division in chlorarachniophytes.

## Results

### Sequences of plastid division protein FtsZD in chlorarachniophytes

**Fig. 1:**
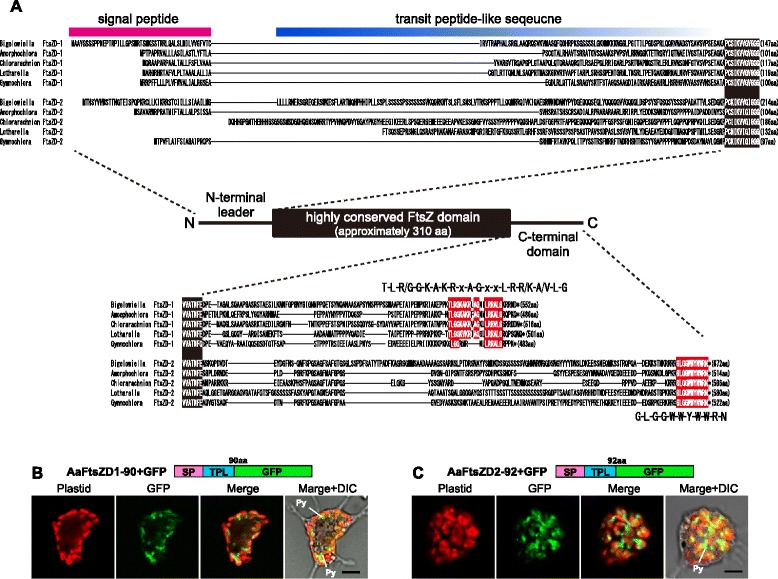
Alignment of N- and C-terminal sequences of chlorarachniophyte FtsZD proteins. **a** The N-terminal leaders are predicted to consist of a signal peptide and a transit peptide-like sequence. Identical amino acids within the highly conserved FtsZ domains are boxed in black, and the C-terminal conserved motifs are highlighted with red boxes. The FtsZD-2 sequences of *Chlorarachnion* and *Lotharella* lack their N-terminal ends. (**b**, **c**) Confocal images of *Amorphochlora amoebiformis* cells expressing GFP fused with the N-terminal leader of AaFtsZD-1 and AaFtsZD-2. The images show red chlorophyll autofluorescence of plastids and green fluorescence of GFP. The pyrenoid (Py) is a part of plastid stroma that has no chlorophyll autofluorescence. The scale bars represent 5 μm

To identify homologous genes of primary plastid division proteins in chlorarachniophytes, we surveyed *B. natans* genomes by reciprocal BLAST with corresponding known sequences of *Arabidopsis thaliana*, *Chlamydomonas reinhardtii*, and *Cyanidioschyzon merolae* [12, 13]. Two homologous genes for FtsZ proteins were found in the *B. natans* nuclear genome as reported previously [43], but the others for ARC6, MinC, MinD, MinE, DRP5B, PDV, ARC3, MCD1, and PDR1 were not detected (Table 1). Two types of *ftsZ* genes, named *ftsZD-1* and *ftsZD-2*, were also identified in RNAseq transcriptome data from four chlorarachniophytes, *Amorphochlora amoebiformis*, *Chlorarachnion reptans*, *Gymnochlora* sp. (CCMP2014), and *Lotharella globosa*. All of these FtsZD proteins were composed of an N-terminal leader, a highly conserved FtsZ domain, and a variable C-terminal region (Fig. 1a). The N-terminal leaders were predicted to be a typical plastid targeting signal consisting of an ER targeting signal peptide and a transit peptide-like sequence (Fig. 1a) by SignalP 3.0 [44] and ChloroP 1.1 [45] programs. To confirm the plastid targeting ability of the N-terminal leaders, we performed GFP-based experiments using the transient transformation system of *A. amoebiformis*. GFP fusion proteins with an N-terminal leader of *A. amoebiformis* FtsZD proteins (AaFtsZD1-N90 + GFP and AaFtsZD2-N92 + GFP) were imported into the plastid stroma (Fig. 1b, c). The C-terminal regions of FtsZD proteins were divergent in sequence and length, whereas their C-terminal ends were mostly conserved among FtsZD-1 and FtsZD-2 proteins; the C-terminal conserved motif of FtsZD-1 is ‘T-L-R/G-G-K-A-K-R-x-A-G-x-x-L-R-R/K-A/V-L-G’ and that of FtsZD-2 is ‘G-L-G-G-W-W-Y-W-W-R-N’ (Fig. 1a).

**Fig. 2:**
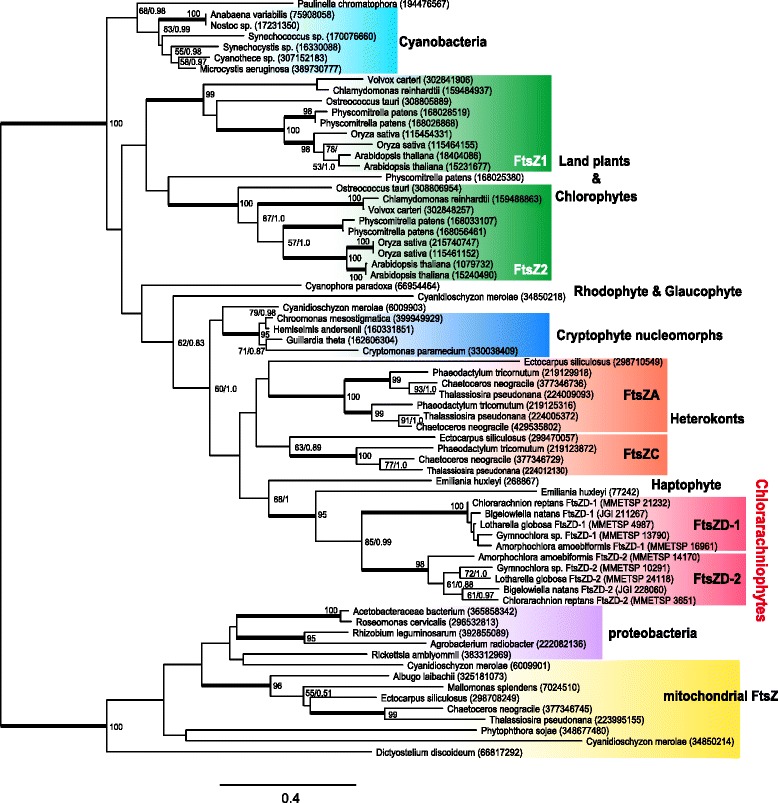
Unrooted phylogenetic tree representing the diversity of eukaryotic and prokaryotic FtsZ. The topology correspond to the best-scoring ML tree as obtained with RAxML. The values at nodes indicate the ML bootstrap supports (BP) and Bayesian posterior probabilities (PP) when they are higher than 50 % and 0.8, respectively. Bold lines correspond to ≥95 % BP and 1.0 PP. GenBank/JGI/MMETSP accession numbers are shown on the right side of species names. The scale bar represents the estimated number of amino acid substitutions per site

Two previous phylogenetic studies reported that chlorarachniophyte *ftsZ* genes appear to be derived from a red algal lineage despite the green algal origin of chlorarachniophyte plastids [43, 46]. To examine the evolutionary process of the two types of *ftsZ* genes in chlorarachniophytes, we reconstructed a phylogenetic tree using prokaryotic and eukaryotic FtsZ sequences with eight chlorarachniophyte FtsZ sequences. The tree indicated the monophyly of chlorarachniophyte *ftsZ* genes, which were closely related to a sequence of the haptophyte *Emiliania huxleyi* bearing a red algal secondary plastid, and the *ftsZD-1* and *ftsZD-2* genes are robustly separated into two clades (Fig. 2). This result strongly suggested that the duplication of *ftsZD-1* and *ftsZD-2* occurred before the divergence of chlorarachniophyte species, whereas the red origin of chlorarachniophyte *ftsZD* genes was not well supported by our analyses.

### Subcellular localization of chlorarachniophyte FtsZD proteins

**Fig. 3:**
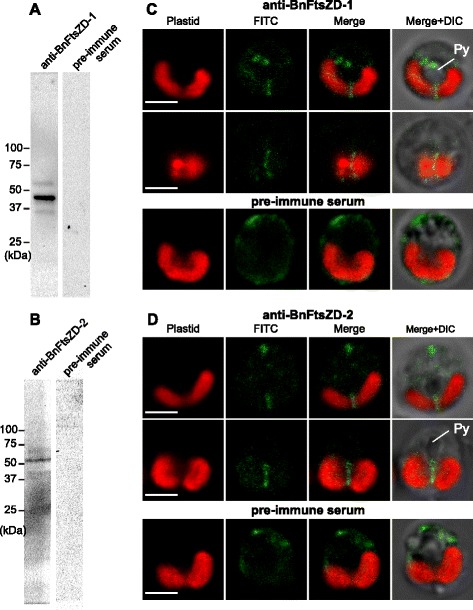
Immunoblot analyses and immunofluorescence microscopy of BnFtsZD-1 and BnFtsZD-2 proteins. Immunoblot analyses of anti-BnFtsZD-1 (**a**) and anti-BnFtsZD-2 (**b**) antibodies against the whole proteins extracted from *B. natans* cells. (**c**, **d**) Confocal images of immunofluorescence labeling of BnFtsZD-1 and BnFtsZD-2 with FITC (green) in *B. natans* cells. Pre-immune serums were used as negative controls. The chlorophyll autofluorescence of plastids is shown by red. Py, pyrenoid. The scale bars represent 2 μm

**Fig. 4:**
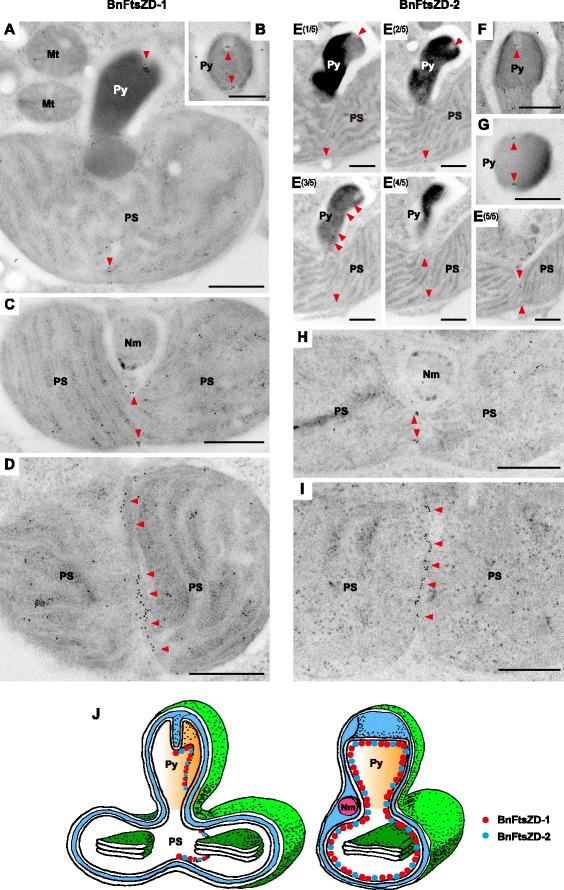
Immunoelectron micrographs of *B. natans* plastids showing localization of BnFtsZD proteins. Images present immunogold localization of BnFtsZD-1 (a to d) and BnFtsZD-2 (e to i). Conjugated gold particles (10 nm) are highlighted with red arrowheads. (**a**) Longitudinal section of plastid stroma (PS) with a projecting pyrenoid (Py). (**b**, **g**) Transverse sections of pyrenoids. (**c**, **h**) Mid-sections of plastid stroma with a nucleomorph (Nm). (**d**, **i**) Transverse sections at the bottom of plastid stroma. (**e**) Serial longitudinal sections of a plastid. (**f**) Longitudinal section of a pyrenoid. The scale bars represent 1 μm. (**j**) Schematic illustration of BnFtsZD localization in the plastid; *red* and *blue* circles correspond to BnFtsZD-1 and BnFtsZD-2, respectively

To determine the detailed subcellular localization of chlorarachniophyte FtsZD proteins, we first examined the full-length of AaFtsZD-1 (486 amino acids) and AaFtsZD-2 (514 amino acids) fused with GFP. Unfortunately their localization was ambiguous and we observed no obvious accumulation of GFP at the plastid division site, probably due to an artifact of high expression of the GFP tagged proteins. Therefore, we generated polyclonal antibodies against *B. natans* FtsZD proteins (anti-BnFtsZD-1 and anti-BnFtsZD-2) to perform immunolocalization experiments. *B. natans* typically has a single plastid in the tiny cell, which has an advantage in ultrastructural studies by electron microscopy. The *B. natans* plastids typically possess a projecting pyrenoid with a plate-like invagination of the two innermost membranes, and a nucleomorph is located near the pyrenoid base [47]. The specificity of two antibodies was examined by immunoblot analyses against the whole proteins extracted from *B. natans* cells; the detected bands were consistent with the predicted size of mature proteins of BnFtsZD-1 (44 kDa) and BnFtsZD-2 (50 kDa) (Fig. 3a, b). We first performed immunofluorescence microscopy. Fluorescein isothiocyanate (FITC) signals were observed at the midpoint of the bilobate plastids in both of the anti-BnFtsZD-1 and anti-BnFtsZD-2 (Fig. 3c, d), and cytoplasmic signals would be a nonspecific background, because such signals were also observed in negative control cells treated with pre-immune serums (Fig. 3c, d). Furthermore, we carried out immunoelectron microscopy using these two antibodies. In both cases, conjugated gold particles were observed near the tip of the shallow invagination of the two innermost plastid membranes within projecting pyrenoids (Fig. 4a, e, f), on opposite sides of the pyrenoid on horizontal sections (Fig. 4b, g), on the innermost membrane in the central narrow part of the bilobate plastids near the nucleomorph (Fig. 4c, h), and in a vertical line on the bottom of the bilobate plastids (Fig. 4d, i). Immunogold signals for both BnFtsZD proteins were almost always detected at the plastid midpoints in observed cells. Taken together, these findings indicated that BnFtsZD-1 and BnFtsZD-2 assembled into a gourd-shaped ring structure on the stromal side of the innermost membrane at the midpoint of bilobate plastids, and the ring always associates with a shallow plate-like invagination of the inner pair of plastid membranes (Fig. 4j). In our electron microscopic observation, electron-dense PD-rings were not detected at the putative plastid division sites where immunogold particles were located.

### Transcription pattern of *ftsZD* genes during the cell cycle

**Fig. 5:**
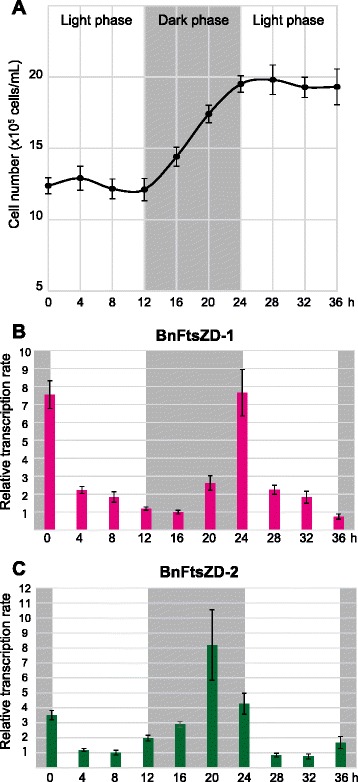
Transcription of *BnftsZD* genes during the cell cycle. **a** Cell densities (cells mL^−1^) in the synchronized *B. natans* culture determined by cell counting; error bars represent standard deviations calculated based on six independent measurements. (**b**, **c**) Relative transcription levels of *BnftsZD-1* and *BnftsZD-2* genes detected using real-time quantitative PCR for a total of 13 time points (4 h intervals for 36 h). Each transcription level was normalized against a transcription level of the reference gene 18S rRNA. Error bars represent standard deviations of triplicate experiments

In unicellular algae with a single plastid, plastid division is generally regulated by the host cell cycle, and transcription of nucleus-encoded plastid division proteins is generally restricted to the S, G2, or M phase, when plastids divide prior to cytokinesis. Transcripts of plastidal *ftsZ* genes are known to accumulate during the S phase of the red alga *C. merolae* [48] and a couple of green algae [18], and during the S/G2 phase of the secondary plastid-bearing diatom *Seminavis robusta* [49]. To clarify the relationship between the timing of *ftsZ* gene expression and host cell division in chlorarachniophytes, we examined the transcription levels of *BnftsZD* genes during the cell cycle using synchronized *B. natans* culture. Cell division was synchronized by pretreatment of continuous light deprivation for 36 h followed by a 12:12 h light:dark cycle; cell division occurred during the second dark phase (Fig. 5a). Total RNA was extracted at 4 h intervals during the second light and dark, and third light phase, and we calculated mRNA transcription levels of *BnftsZD-1* and *BnftsZD-2* using real-time quantitative PCR (RT-qPCR). Transcription levels of both genes increased soon after cell division in the late dark phase (Fig. 5b, c). The transcription level of *BnftsZD-1* reached a peak at the end of the dark phase corresponding to the host M/G1 phase, whereas the peak of *BnftsZD-2* was 4 h before that of *BnftsZD-1*. Both *BnFtsZ* genes appeared to be regulated by the host cell cycle. Unlike other unicellular algae, however, the transcription pattern of chlorarachniophyte *ftsZD* genes is not consistent with the timing of plastid division that occurs before the cytokinesis. When the mRNA transcription ratio between *BnftsZD-1* and *BnftsZD-2* was simply calculated by Ct values of RT-qPCR at their peaks (24 h and 20 h for *BnftsZD-1* and *2*, respectively), the maximum transcription level of *BnftsZD-1* was 2.3-fold higher than that of *BnftsZD-2*. This suggests that two BnFtsZD proteins might exist in the plastids in different molecular amounts.

## Discussion

### Prospective function of FtsZD proteins in chlorarachniophytes

One previous ultrastructural study of the chlorarachniophyte *B. natans* has shown that divisions of the plastid and the nucleomorph take place in an early stage of cell division, whereas partial bisection of the pyrenoid with an invagination of the two innermost membranes appears to start very soon after cell division [47]. Therefore, a shallow membrane invagination is almost always observed within the pyrenoids by electron microscopy. This infers that the partial membrane constriction would be achieved by a mechanism that is independent of plastid division. Our immunolocalization results indicate that the two FtsZD proteins of *B. natans* accumulate to form a gourd-shaped ring structure associated with the membrane invagination of projecting pyrenoids (Fig. 5j). This suggests that the FtsZD proteins are involved in the partial constriction of the inner pair of plastid membranes, which is supported by the finding that the expression patterns of *BnftsZD* genes are consistent with the timing of formation of the membrane invaginations. Interestingly, the membrane constriction somehow arrests on the way, and the FtsZD proteins are kept in a ring-like structure along the membrane invagination. However, it remains unknown how the membrane constriction is controlled and when the plastid stroma is completely divided.

The projecting pryrenoids of chlorarachniophyte plastids have a remarkable morphological diversity in membrane invaginations among genera, which is utilized for their taxonomy. The pyrenoids of *Bigelowiella* and *Norrisiella* have a shallow invagination of the two innermost plastid membrane (the depth is less than half the pyrenoid’s height) [50], whereas *Lotharella*, *Amorphochlora*, and *Chlorarachnion* pyrenoids possess a deep invagination that reaches the plastid stroma [51]. It is expected that variable degrees of Z ring constriction might cause the morphological diversity of chlorarachniophyte plastids. Unlike other chlorarachniophytes, *Gymnochlora* have many single-membraned tubular invaginations in the pyrenoids [52], implying that this genus might exhibit lineage-specific behavior of FtsZD localization.

### Functional divergence of two FtsZ families

Although cyanobacteria possess a single gene for FtsZ protein, multiple types of plastidal FtsZ proteins have been reported in plants and algae; Viridiplantae (land plants and green algae) have two phylogenetically distinct types of FtsZ families named FtsZ1 and FtsZ2, red algae have FtsZA and FtsZB, and secondary plastid-bearing heterokonts possess FtsZA and FtsZC [53]. Two distinct plastidal FtsZ families emerged by multiple duplication events of an *ftsZ* gene originally derived from a cyanobacterium in each common ancestor of Viridiplantae, red algae, and heterokonts [53]. Green lineage FtsZ2 and red lineage FtsZA as well as cyanobacterial FtsZ contain a highly conserved sequence “D/E-I/V-P-x-F/Y-L” in their C-termini, the “C-terminal core domain”, whereas other FtsZ1, FtsZB, and FtsZC proteins lack this domain [53, 54]. This suggests that one of two FtsZ families have lost the C-terminal core domain after the duplication events. In the model plant *A. thaliana*, the C-terminal core domain of FtsZ2 mediates a specific interaction with the transmembrane protein ARC6 localized at the plastid division site to tether the Z ring to the inner plastid membrane [19]. We revealed that chlorarachniophytes also have two distinct FtsZ families, FtsZD-1 and FtsZD-2, that emerged by gene duplication in a common ancestor of chlorarachniophytes (Fig. 2). Both families carry chlorarachniophyte-specific C-terminal conserved motifs, T-L-R/G-G-K-A-K-R-x-A-G-x-x-L-R-R/K-A/V-L-G within FtsZD-1 and G-L-G-G-W-W-Y-W-W-R-N within FtsZD-2, which are different from the C-terminal core domain of FtsZ2 and FtsZA. This suggests that chlorarachniophyte FtsZD-1 and FtsZD-2 independently acquired the unique C-terminal motifs after gene duplication. Because of the absence of homologous gene for ARC6 in chlorarachniophyte genomes, the C-terminal conserved motifs are not involved in the ARC6-medicated membrane tethering, and the functional importance of the C-terminal conserved motifs remains uncertain.

Genetic complementation experiments demonstrated that *Arabidopsis* FtsZ1 and FtsZ2 were functionally non-redundant in plastid division [55]. Their functional divergence is partly explained by the facts that only FtsZ2 has a specific interaction with the transmembrane protein ARC6 [19] and turnover of the FtsZ1 filaments is faster than that of FtsZ2, suggesting that FtsZ1 facilitates Z ring remodeling [56]. Furthermore, it has been reported that *Arabidopsis* FtsZ1 and FtsZ2 maintain a constant 1:2 ratio in whole mature leaves [57], and that they are able to form heteropolymers at variable ratios in vitro [58]. In this study, we found that the two types of *B. natans ftsZ* genes, *BnftsZD-1* and *BnftsZD-2*, showed slightly different transcription patterns in the cell cycle, and the maximum transcription level of *BnftsZD-1* was predicted to be approximately 2-fold higher than that of *BnftsZD-2*. These findings lead us to infer that the two FtsZ families of chlorarachniophytes also exhibit functional divergence similar to *Arabidopsis* FtsZ families. The earlier transcription of *BnftsZD-2* than *BnftsZD-1* raise the possibility that BnFtsZD-2 plays a role in an early stage of Z ring assembly and positioning.

### Evolution of the plastid division machinery in secondary plastids

In land plants, the Z ring assembly is restricted to the plastid midpoint by several division-site positioning factors, ARC3, MCD1, MinD, and MinE [54]. Homologous genes for cyanobacteria-derived Min proteins have also been found in a glaucophyte and green algae, as well as secondary plastid-bearing algae (Table 1). Therefore, a portion of the Z ring positioning mechanism appears to be widely conserved in both primary and secondary plastids. However, the chlorarachniophyte *B. natans* completely lacks all genes for the plant-like Z ring positioning factors, similar to the red alga *C. melorae*. Despite the absence of canonical positioning factors, FtsZ proteins are able to assemble at the plastid midpoint in both algae, suggesting that they have independently evolved a currently unknown mechanism for Z ring positioning.

The division complex of primary plastids generally consists of two different GTPase proteins, FtsZ and DRP5B, localizing to ring-like structures on the stromal side of the inner membrane and the cytoplasm side of the outer membrane, respectively. Homologous genes for FtsZ and DRP5B have been found in some secondary plastid-bearing algae, heterokonts and a haptophyte, and these proteins are assumed to function in the constriction of the inner pair of four plastid membranes [11]. However, no genes for DRP5B have been identified in genomes of *B. natans* and the cryptophyte *Guillardia theta* [32]. Previous studies have reported that *drp5B* (*arc5*) mutants of *A. thaliana* and the moss *Physcomitrella patens* exhibit defects in complete plastid constriction, resulting in enlarged, dumbbell-shaped plastids [25, 59, 60]. However, each cell of the *drp5B* mutants contains a small number of plastids, indicating that the plastids can still divide into daughter plastids without DRP5B. Therefore, it is possible that the two innermost membranes of secondary plastids are constricted by an FtsZ-dependent but DRP5B-independent mechanism in chlorarachniophytes and cryptophytes, like the plant *drp5B* mutants. Furthermore, the absence of DRP5B might be related to the partiality of membrane constriction in chlorarachniophyte plastids.

In some secondary plastids, constriction of the outer pair of plastid membranes takes place behind that of the inner pair [61]. Although it is not known how the outer pair of membranes divide, the constriction is performed by a mechanism that is distinct from the mechanism for the inner pair. In an apicomplexan parasite, the apicomplexan-specific dynamin-related protein DrpA presumably localizing on the surface of four membrane-bound apicoplasts is essential for apicoplast division [38]. It may be possible to infer that a lineage-specific dynamin-like protein is involved in constriction of the outer pair of plastid membranes in other secondary plastid-bearing organisms. The *B. natans* genome is found to encode two highly divergent dynamin-like proteins [GenBank ID: 113201831 and 113201829], and future studies on these proteins will provide further insights into our findings.

## Conclusions

We characterized two plastid division proteins, named FtsZD-1 and FtsZD-2, in chlorarachniophyte algae possessing secondary plastids. These two distinct FtsZ families emerged by gene duplication in a common ancestor of chlorarachniophytes. Both FtsZD proteins assemble to a ring-like structure at the midpoint of bilobate plastids with a projecting pyrenoid. The ring was associated with a shallow plate-like invagination of the inner pair of plastid membranes. Transcription level of *B. natans ftsZD* genes was periodically increased soon after the cell division, which is consistent with the timing of the formation of the membrane invagination. Our findings suggest that chlorarachniophyte FtsZD proteins are involved in partial constriction of the inner pair of plastid membranes, but not in the whole process of plastid division. At present, it remains uncertain how the outer pair of plastid membranes is constricted in secondary plastid-bearing algae. We need further investigations to determine the whole system of secondary plastid division.

## Methods

### Chlorarachniophyte cultures

*B. natans* (CCMP2755) and *A. amoebiformis* (CCMP2058) cells were grown at 22 °C under white illumination (80–100 μmol photons m^−2^ s^−1^) on a 12:12 h light:dark cycle in ESM medium. Synchronization of *B. natans* cell division was achieved as previously described [62].

### Homology searches of plastid division proteins

Genes for plastid division proteins (FtsZ, ARC6, MinC, MinD, MinE, DRP5B, PDV, ARC3, MCD1, and PDR1) were identified using whole genome data of land plants and several algae in previous studies [13, 18]. To update the information, homologous genes for plastid division proteins were searched in the whole genomes of secondary plastid-bearing algae (*Thalassiosira pseudonana*, *Phaeodactylum tricornutum*, *Aureococcus anophagefferens*, *Emiliania huxleyi*, *Guillardia theta*, *Toxoplasma gondii*, and *Bigelowiella natans*), based on reciprocal BLASTp with e-value cutoff of 1e^−10^ using corresponding known sequences of *Arabidopsis thaliana*, *Chlamydomonas reinhardtii*, and *Cyanidioschyzon merolae*. The genome sequences were obtained from the GenBank and the DOE Joint Genome Institute (JGI) site.

### Phylogenetic analyses

Chlorarachniophyte *ftsZ* genes were identified from the genome sequence of *B. natans* [40] and Illumina-based transcriptomes of four chlorarachniophytes, *A. amoebiformis*, *C. reptans*, *Gymnochlora* sp., and *L. globosa*, using tBLASTn homology search with corresponding sequences of *A. thaliana*, *C. reinhardtii*, and *C. merolae*. The transcriptome analyses were performed by the Marine Microbial Eukaryote Transcriptome Sequencing Project [63], and the sequence data are available from the iMicrobe website (http://data.imicrobe.us/project/view/104) under sample IDs: MMETSP 0041, 0042, 0109, and 0110. Phylogenetic trees were constructed with 60 sequences of prokaryotic and eukaryotic FtsZ proteins retrieved from GenBank and 10 chlorarachniophyte sequences. The sequences automatically aligned by the L-INS-i method of the MAFFT package [64], and poorly aligned positions were manually inspected with MEGA6 [65]. Phylogenetic analyses were performed using Maximum-likelihood (ML) and Bayesian methods with the final dataset including 315 unambiguously aligned amino acid positions from 70 taxa. ML analyses were carried out using RAxML v8.1.15 [66] with the LG + GAMMA model selected as the best-fit model by Aminosan [67]. The best-scoring ML tree was determined in multiple searches using 10 distinct randomized maximum-parsimony trees, and statistical support was evaluated by 1,000 bootstrap replicates. Bayesian analyses were performed using MrBayes v3.1.2 under the LG + GAMMA model [68]. Metropolis-coupled Markov Chain Monte Carlo was run from a random starting tree for 1,000,000 generations, sampling every 1,000 cycles. Three heated and one cold chains were run simultaneously and the initial 380,000 cycles were discarded as burn-in. Node posterior probabilities were calculated from the remaining 620 trees.

### Localization analyses of GFP fusion proteins

Total RNA was extracted from *A. amoebiformis* cells using TRIzol reagent (Invitrogen), and cDNA was synthesized using SuperScript II Reverse Transcriptase (Invitrogen) with an oligo (dT) primer. To construct plasmids encoding GFP fusion proteins, cDNA fragments encoding the N-terminal leaders of AaFtsZD-1 (90 amino acids) and AaFtsZD-2 (92 amino acids) were amplified by PCR with specific primer sets (see Additional file 1), and each fragment was inserted between *Hin*dIII and *Nco*I sites of pLaRGfp + mc vector [69] that carried the promoter of a rubisco small subunit gene. To analyze the subcellular localization of GFP fusion proteins, *A. amoebiformis* cells were transformed with each plasmid using a Biolistic PDS-1000/He particle delivery system (Bio-Rad) as described previously [70]. Twenty-four to 48 h after transformation, the transient transformed cells expressing reporter genes were observed under an inverted Zeiss LSM 510 laser scanning microscope (Carl Zeiss) with the single-track mode.

### Preparation of antibodies

Polyclonal antibodies against *B. natans* FtsZD proteins were raised in rabbits using the respective recombinant proteins corresponding to their mature proteins of 210 amino acids (from 256 to 444 residue of BnFtsZD-1 or from 326 to 514 residue of BnFtsZD-2) by BioGate Co., Ltd (Gifu, Japan). Amplified cDNA fragments encoding these BnFtsZD mature proteins were cloned into pET28 expression vectors, and 6xHis fusion polypeptides were expressed in Rosetta 2 (DE3) *Escherichia coli* cells (Novagen), according to the manufacture’s instruction. The recombinant proteins were purified with His GraviTrap columns (GE Healthcare) under a denaturing condition with 8 M urea, followed by an overnight dialysis against phosphate-buffered saline (PBS). Polyclonal rabbit immunoglobulin G (IgG) antibodies were purified using HiTrap Protein A HP columns (GE Healthcare). The specificity of antibodies was tested by immunoblot analyses with the total proteins extracted from *B. natans* cells. The proteins were electrophoresed on a 12 % SDS polyacrylamide gel, and blotted to a PVDF membrane using a Trans-Blot Turbo Transfer System (Bio-Rad). Immunoblotting was performed by an iBind Western system (Life Technologies) with each of anti-BnFtsZD antibodies/pre-immune serums diluted 1:500, followed by horseradish peroxidase (HRP)-conjugated secondary antibodies at a dilution of 1:10,000. The signals were detected with ECL Prime Western Blotting Detection Reagent (GE Healthcare) and a ChemiDoc MP System (Bio-Rad).

### Immunofluorescence and Immunoelectron microscopy

For immunofluorescence microscopy, *B. natans* cells were fixed for 1 h at room temperature in 4 % paraformaldehyde/0.25 M sucrose in PHEM buffer (60 mM PIPES, 25 mM HEPES, 10 mM EGTA, 2 mM MgCl2; pH 7.4). Fixed cells were transferred onto poly-L-lysine coated coverslips, and rinsed with PBS. Permeabilization of cell membranes was performed with 5 % tween 20 in PBS for 10 min; the detergent Triton X-100 was not useful because the chlorophyll autofluorescence was removed even in a low concentration of 0.01 %. After blocking with 3 % skim milk/1 % normal goat serum in PBS for 30 min, the cells were treated for 2 h at room temperature with respective anti-BnFtsZD antibodies/pre-immune serums diluted to 1:50 in PBS. The cells were washed by PBS, and then the primary antibodies were labeled for 1 h with a FITC-conjugated anti-rabbit IgG antibody (Sigma: F9887) diluted to 1:100 in PBS. The cells were mounted using a SlowFade Diamond antifade, and observed under an inverted Zeiss LSM 510 laser scanning microscope (Carl Zeiss).

For immunoelectron microscopy, specimens were prepared using a modified protocol of cryofixation and freeze substitution described previously [71]. Cryofixed *B. natans* cells were incubated in cooled 100 % ethanol (−80 °C) for 2 days, and finally embedded in LR White resin. Ultrathin sections of polymerized blocks were created by a Leica ultramicrotome EM UC6, and collected onto Formvar-coated copper grids. Immunogold labeling was performed with respective anti-BnFtsZD antibodies at a dilution of 1:10 in PBS, and a gold-conjugated anti-rabbit IgG secondary antibody (Sigma: G7402) diluted to 1:20. The ultrathin sections were stained with uranyl acetate for 10 min, and observed under a Hitachi H7650 transmission electron microscope at 80 kV.

### Real-time quantitative PCR

*B. natans* cells were harvested from 100 mL synchronized cultures at 4 h intervals for 36 h, and the cell number was monitored with a Fuchs-Rosenthal hemocytometer. Total RNA was extracted from 13 time points using TRIzol reagent (Invitrogen), and cDNA was synthesized using a ReverTra Ace qPCR RT Kit (Toyobo) according to the manufacture’s protocols. Primers for quantification of *BnftsZD-1* and *BnftsZD-2* were designed using the Primer3Plus online software (primer sequences were listed in Additional file 1), and a previously reported primer set of 18S rRNA [62] was used for normalization. RT-qPCR was performed using a Thermal Cycler Dice Real Time System II (Takara) under the following conditions: 1 μL of cDNA, 0.4 μM of each primer, 12.5 μL of SYBR Premix Ex Taq II (Takara), and DNase/RNase-free water up to 25 μL. The cycling conditions comprised 3 min of denaturation at 95 °C, followed by 40 cycles of 10 s at 95 °C and 30 s at 60 °C, and a melting curve program for detection of nonspecific products. Relative changes in gene expression were calculated by the ΔΔCT method [72] in different time points.

## Availability of supporting data

Nucleotide sequences used in this study can be obtained from GenBank (National Center for Biotechnology Information), the JGI genome portal site of *Bigelowiella natans* (http://genome.jgi.doe.gov/Bigna1/Bigna1.home.html), and the *Cyanophora paradoxa* Genome Project site (http://cyanophora.rutgers.edu/cyanophora/home.php), and the iMicrobe website (http://data.imicrobe.us/project/view/104).

## Additional file

Additional file 1:
**Primer sequences for plasmid construction (Table S1) and primer sequences for** **RT-qPCR** **experiments (Table S2).** (PDF 120 kb)

## Abbreviations

ER: endoplasmic reticulum
GFP: green fluorescent protein
FITC: fluorescein isothiocyanate
RT-qPCR: real-time quantitative PCR
IgG: immunoglobulin G
