# Supplementary material for: Prospective function of FtsZ proteins in the secondary plastid of chlorarachniophyte algae
Source: BMC Plant Biol. 2015 Nov 10;15:276. doi: 10.1186/s12870-015-0662-7 (PMC4641359; doi:10.1186/s12870-015-0662-7)
Supplement: Additional file 1: — Primer sequences for plasmid construction (Table S1) and primer sequences for RT-qPCR experiments (Table S2). (PDF 120 kb) [file 12870_2015_662_MOESM1_ESM.pdf]

Table S1. Primer sequences for plasmid construction

Fragment name	Sequence (5' to 3')	note
AaFtsZD1-90	ACCAAGCTTATGCCAACTCCAGCCC	Forward (HindIII)
	ACACCATGGGAGCCTTACCCGCTTCCGA	Revers (NcoI)
AaFtsZD2-92	ACCAAGCTTATGTCTGCTGTGGCTCG	Forward (HindIII)
	ACACCATGGAGCTATAGTTGATGTCATC	Revers (NcoI)

Table S2. Primer sequences for real-time quantitative PCR

Fragment name	Sequence (5' to 3')	note
BnftsZD-1	GTCGAGTTCTGGGCAGTCAATAC	Forward
	ATTGTAGGTTTCGCTCCTGCTC	Reverse
BnftsZD-2	AATGGCAGCTATCACGTCTCC	Forward
	CTCTGCGACACGATTGACC	Reverse
Bn18rRNA	TGCCAGGCGATAGTTCATTC	Forward
	TTGGATGTGGTAGCCGTTTC	Reverse
